# Decolonizing the digital landscape: the role of technology in Indigenous language revitalization

**DOI:** 10.1177/11771801211037672

**Published:** 2021-08-11

**Authors:** Paul J Meighan

**Affiliations:** Department of Integrated Studies in Education (DISE), Faculty of Education, McGill University, Canada

**Keywords:** decolonizing technology, Indigenous language revitalization, online, technology

## Abstract

Due to colonization and imperialism, Indigenous languages continue to be threatened and endangered. Resources to learn Indigenous languages are often severely limited, such as a lack of trained or proficient teachers. Materials which follow external standards or Western pedagogies may not meet the needs of the local community. One common goal for Indigenous language revitalization initiatives is to promote intergenerational language transmission and use in multiple social domains, such as the home. Could the use of technology assist in Indigenous language revitalization? And what would be its role? This article, emerging from ongoing research, aims to synthesize some key takeaways on the role of digital and online technologies in Indigenous language revitalization over the past three decades since the foundation of the World Wide Web in 1989. The article highlights how Indigenous communities, content creators, scholars and visionaries have contributed to an ongoing decolonization of the digital landscape.

## Positionality

Halò a chàirdean. Is mise Pòl Miadhachàin-Chiblow. ‘S e Gàidheal a th’ annam. Tha mi à Glaschu, ann an Alba. Hello, friends. My name is Paul Meighan-Chiblow. I’m a Gael from Glasgow, Scotland.

My research focuses on Indigenous language revitalization and is informed by experiences as a Gàidheal (Scottish Gael) in Glasgow, Scotland where, for example, my endangered mother tongue, Gàidhlig (Scottish Gaelic), was not available to me in the educational system. Members of my family and older generations recall being beaten for speaking it in school, and Gàidhlig, spoken for more than 1500 years in Scotland, is still not recognized as an “official” language in the United Kingdom. My motivation for equitable education and language revitalization has continued to grow after meeting my Anishinaabe (Indigenous Peoples of the Great Lakes region of Turtle Island, also known as North and Central America) husband in Scotland. I learned more about the devastating impacts of colonialism on the Indigenous Peoples of Turtle Island. We talk frequently about the importance of reclaiming and speaking our languages, languages which have been oppressed and pushed to the verge of extinction by centuries of colonial governments and educational policy. We want to speak our languages in our home and with our future children.

I am grateful to currently reside with my husband in T’karonto (Toronto), the traditional lands and territory of many nations including the Mississaugas of the Credit, the Anishinaabeg, the Chippewa, the Haudenosaunee and the Wendat Peoples and now home to many diverse First Nations, Inuit and Métis Peoples. We acknowledge the waters, animals, plants and all more than human entities of Turtle Island and our responsibilities to them. Miigwetch (thank you in Anishinaabemowin, the language of the Anishinaabe Peoples) and tapadh leibh (thank you in Gàidhlig).

## Introduction

Due to colonization and imperialism, Indigenous languages continue to be threatened and endangered (Chiblow & Meighan, 2021). Resources to learn Indigenous languages are often severely limited, such as a lack of trained or proficient teachers. Materials which follow external standards or Western pedagogies may not meet the needs of the local community. One common goal for Indigenous language revitalization (ILR) initiatives is to promote intergenerational language transmission and use at home. Could the use of technology assist in ILR? And how do we define technology and its role?

Technology is much more than just machines. Technology is the result of practical applied knowledge, skills and networks which are continually evolving, fluid and context-dependent (Silverstone, 2005). In other words, technology is not neutral and is the extension of the knowledge and belief system which has led to its creation (Strate, 2012). Examples of technology include writing systems, the pencil, the wampum belt, mass media, television and, more recently, online and digital technologies, such as the Internet and cellphones.

A fundamental issue that needs to be taken into consideration when discussing the role of technology in ILR is identifying which or whose knowledge system is being enacted. Who created the website? What is its purpose? How is data being shared or stored online? These questions and concerns are particularly crucial when it comes to discussing Indigenous languages and cultures which have been disprivileged and disenfranchised by imperialistic, capitalistic and colonial knowledge systems (Battiste, 2002; Macedo, 2019). Pool (2016) underscores, “for their colonising mission, imperialists imported data methodologies, smugly assuming that epistemologies other than Euro-North American ones were inferior. This view still haunts the wider society’s acceptance of information systems now being generated by Indigenous scholars” (p. 62).

This article, emerging from ongoing research, aims to synthesize some key takeaways on the role of technology in ILR over the past three decades and highlight how Indigenous Peoples have contributed to an ongoing decolonization of the digital landscape. The article is non-exhaustive in that the context and discussion largely focus on the rapidly evolving landscape and influential proliferation of digital and online technologies in the past three decades since the creation of the World Wide Web in 1989.

## Methods

A variety of academic and grey literature, such as reports, policy literature and government documents, from the past three decades have been synthesized for the purposes of this conceptual review article. Additional sources, such as websites and online newspapers, have also been implemented to inform the discussion and complement the academic and grey literature.

A decolonizing approach, informed by Indigenous research methodologies and epistemological frameworks (Battiste, 2002; Smith, 2012), to reviewing and synthesizing the literature has been taken. This approach decenters search results determined by colonial and Western library knowledge organization systems alone. For example, algorithms or metadata that classify Indigenous knowledge as folklore (White, 2018). Each source and literature item were selected based on fulfilling at least three of the following search criteria: colonization or decolonization; Indigenous or colonial education; Indigenous or Western technology and social media; Indigenous language revitalization inquiry and technological initiatives; Indigenous methodologies and methods. Citation and reference list snowballing was also used to include additional, relevant peer-reviewed, grey and online literature.

## The types, stages and applications of technology

Technology has been present for thousands of years, from power technology, such as the use of fire during the Old Stone Age, all the way through to our current day use of cellphones and social media. During this time, technology has evolved through varying stages, which have been summarized non-exhaustively in Table 1. The purpose of the table is to identify technological and relational trends in the past, present and future and to highlight the rapid development of digital and online technologies over the past three decades to this day.

**Table 1.** Types, stages and evolution of technology use.

Types and stages of technology	Examples	Relationship	Evolution
1. Facilitation technologies	Crockery, pots, guns, agricultural machinery and tools	Individual and group → local environment	*Facilitation*
2. Communication technologies	Writing systems (e.g., pictographs), writing implements, mass media (e.g., television), telephone, typewriter, computer	Individual, group and state → mass audience	*Communication*
3. Web 1.0 Digital and online technologies (~1990–2005)	Digital cellphone, multimedia (e.g., DVD, CD-ROM)	State and group → mass audience	*Digital Information*
4. Web 2.0 Digital and online technologies (~2005–2015)	Social media, smartphones, video games, the Cloud, broadband	State and group ↔ mass audience; Peer-to-peer (P2P)	*Digital Negotiation*
5. Web 3.0 Digital and online technologies (~2015–present)	Augmented reality (AR), virtual reality (VR), blockchain	P2P; Peer ↔ mass audience	*Digital Creation*
6. Semantic technologies: (The future)	Internet reality, artificial intelligence (AI), 3D and 4D avatars	Technology ↔ human audience ↔ environment	*Digital Simulation*

Technology has been grouped into six types which have been listed in approximate chronological stages with examples in Table 1: (1) Facilitation technologies; (2) Communication technologies; (3) Web 1.0 Digital and online technologies (~1990–2005); (4) Web 2.0 Digital and online technologies (~2005–2015); (5) Web 3.0 Digital and online technologies (~2015–present); and (6) Semantic technologies.

These categorizations are not indicative of all technological developments and are not neat or concrete historical or chronological boundaries. Instead, they serve as a basis to exemplify how technology has been viewed and utilized in dominant Western ideals of technological progress. Underscoring the dominant Western capitalistic worldview is particularly important here as the World Wide Web was created by Western people for a Western audience. For example, Tim Berners-Lee, the inventor of the World Wide Web, envisaged “universality” and “dictated the monolingual [English] design of the web” (Kelly-Holmes, 2019, p. 28). The Web, however, is not monolithic or linear in terms of development and is still evolving. The notions of Web 1.0, 2.0 and 3.0, “although important when analysing the political economy of the Web” (Barassi & Treré, 2012, p. 1269), have limitations and are cultural constructs influenced by Western business rhetoric. They often carry “generalized understandings about the social uses of technology” (Barassi & Treré, 2012, p. 1281).

Table 1 also illustrates the relationship of technology between humans and interactants and the socio-technological evolution from *Facilitation*, *Communication*, *Digital Information*, *Digital Negotiation*, *Digital Creation*, to *Digital Simulation*. The relationship and evolution of technology is crucial to undertake a more holistic and nuanced assessment of technology’s social impact on ILR and better understand who is enacting technology and for whom. The continually evolving Internet, or Web, is a complex socio-technical environment with multiple uses dependent on social contexts and relationships (Barassi & Treré, 2012).

This article will focus on the role of digital and online technologies in ILR during the Web 1.0, 2.0 and 3.0 eras. The purpose is to track what developments there have been in technology’s social role and what these trends signify for a decolonized, more than Western, digital landscape and future.

## Digital and online technology use in ILR initiatives

### Technology use in the Web 1.0 period (~1990–2005)

During the Web 1.0, there were several examples of ILR initiatives which used the affordances of the new World Wide Web and digital technologies, such as the desktop computer and the CD-ROM.

One of the first ILR initiatives to utilize the potential of the World Wide Web and Communication Technologies was Te Wahapu (The Estuary). Te Wahapu was a computer-based communications system created in 1990, focused on the revitalization of the Maori language in New Zealand to “symbolize the integration of high technology with Maori concerns and interests . . .[and] convey the message that ‘English has no monopoly when it comes to making use of advanced technology’” (Benton, 1996, p. 189).

Another example is Leoki (Powerful Voice), an electronic bulletin board system established in 1993 and delivered entirely in the Hawaiian Indigenous language (Warschauer, 1998). Leoki provided “online support for Hawaiian language use in the immersion schools and the broader community” (Warschauer, 1998, p. 142). Leoki facilitated the creation of materials which were both culturally responsive and in the language.

Revitalization and reclamation strategies during the Web 1.0 era have involved creating spoken and written dictionaries and audio- or video-recording Elders speaking their Indigenous language. The web-based resource FirstVoices, founded in British Columbia, is an example of how technology has been utilized by First Nations’ communities in Canada to document, archive and learn Indigenous languages using text, sound and video clips (First Peoples’ Cultural Foundation, 2003). Users can to this day interact on the site, which includes an archive, chat facility, games, videos, storybooks and a language tutor.

Interactive CD-ROMs and other types of multimedia have also been used for ILR during the Web 1.0 era. In Alaska, the Lower Kuskokwim School District produced a bilingual CD-ROM in English and Yup’ik, a central Alaskan language, for the traditional story *How the Crane got Blue Eyes* (Cazden, 2002). A computerized database in the Tlingit language, spoken by the Tlingit people of southeast Alaska and western Canada, had historical information and a talking map in Tlingit and English (Cazden, 2002). The Unipkaaqtualiurut Project and the Uggianaqtuq CD-ROM recorded Elders and documented environmental knowledge in Inuktitut, an Inuit language (Gearheard, 2005). In central California, interactants could view performances of traditional and contemporary verbal art in the Mono language on the Taitaduhaan (Our Language) CD-ROM and select an option to view with translation into English (Kroskrity & Reynolds, 2001). Other examples of multimedia ILR initiatives from across the globe include a modern-day television soap opera in Scottish Gaelic (Cormack, 1994) and a CD-ROM for adolescents about ice hockey in Ojibwe (Williams, 2002).

### Key takeaways and insights on the role of technology in the Web 1.0 Era

The examples of ILR initiatives above have been fundamental for making Indigenous voices heard and represented across the globe during the beginnings of the digital age. The ILR technology-enabled initiatives enabled Indigenous communities to “cut out the middle people . . . and speak directly to their audience” (Jopson, 1997, para. 10).

As Table 1 illustrates, this era was characterized by the evolution of a largely unilateral transfer of *Digital Information*. Information pertaining to Indigenous languages, cultures or communities was placed on the Internet by a group, community or state, without the broader input of those who were using the materials. Despite many Web 1.0 initiatives being coined interactive, such as the ability to listen, view or click on materials, there was a lack of co-creation of knowledge or user input on material development. The majority of the initiatives were examples of *low-tech* (Galla, 2009) projects based on one sensory mode: in this case, output over input. Looking in more detail at who creates endangered language websites and the level of knowledge co-creation, Buszard-Welcher (2001) finds that 38% of the 50 sites on “Native American or Canadian” Indigenous languages belonged to groups and only four of those were created by a “Tribal” member or official organization (p. 332).

Some of the CD-ROMs lacked cultural context, such as in the case of the Yup’ik language and culture (Cazden, 2002). For instance, Indigenous words were placed on the CD-ROM without a literal or faithful translation which could transmit valuable knowledge about the origins of a word or phrase and the local ecosystem. Leonard (2001) gives an example of Vichingadh Ethog (Yellow Pond Lily) in Deg Xinag, an Alaskan language. A more faithful translation would be “Muskrat’s Plate” (Leonard, 2001, p. 4). Leonard remarks, “For a beginning language learner, literal translations provide a great deal of fascinating cultural information and further impetus for investigation into one’s own culture” (p. 4). Much of the material in the Web 1.0 era for ILR did not go beyond the “word or phrase level” (Galla, 2009, p. 176). Much of the content was primarily bilingual or framed in the dominant language, English, and the Western worldview. Galla (2016) remarks on materials from the 1990s that “a significant challenge that language instructors of endangered languages face is a lack of textbooks, pedagogical, culturally relevant, appropriate, and authentic materials that depict the language and culture in a non-stereotypical way” (p. 1146).

The cost of developing and creating materials and software, filming, recording and purchasing hardware, such as desktops and other multimedia tools, were very costly and involved considerable amounts of time (Kroskrity & Reynolds, 2001). Access to the newly created Internet and hardware or software was limited to certain areas or people who had the ability to connect and also afford the costs of being online (Carpenter et al., 2017). This *digital divide* was more pronounced for some communities, and many who could have benefitted from the technological innovations of this period did not have as many chances to fully participate.

Technology in the Web 1.0 era did offer much potential for ILR and also had an additional “cool” element (Buszard-Welcher, 2001, p. 337). This element can appeal particularly to the younger Indigenous generation and help restore prestige and pride in Indigenous languages and cultures (Buszard-Welcher 2001). Technology, despite not yet being fully dialogic in terms of co-creation of knowledge, was a means of interaction among language activists. Email lists and the like enabled platforms for sharing Indigenous innovations, aspirations and concerns across different website groups (Grenoble & Whaley, 2006; Warschauer, 1998). Technology connected language activists both within, across and outside of Indigenous communities, fostered relationships across the globe, and was a crucial “key motivator in the sense they are ‘not going it alone’” (Grenoble & Whaley, 2006, p. 190).

### Technology use in the Web 2.0 and Web 3.0 eras (~2005–present)

ILR initiatives have begun to build upon the strategies incorporated during the Web 1.0 era and take advantage of new advances in digital and online technologies during the Web 2.0 and Web 3.0 eras. In dominant Western business rhetoric, Web 2.0 era is characterized by increased user participation or collaboration (Barassi & Treré, 2012). Examples of Web 2.0 digital and online technologies are faster broadband Internet speeds; P2P sharing and creation, such as Wikipedia; social media, such as Facebook, YouTube and Twitter; and the smartphone (see Table 1). The emerging Web 3.0 era is viewed as having increased user creation, cooperation (Barassi & Treré, 2012) and a decentralization, localization and democratization of power. Examples of Web 3.0 technologies include blockchain distributed ledger technology, geolocation, and augmented reality (AR) or virtual reality (VR).

The Web 2.0 and 3.0 periods will be discussed together. Forms of technology use between 2005 and the present day have involved cross-over elements and interplay which are still emerging, can be categorized as both Web 2.0 and Web 3.0, and do not neatly fit a chronological timeframe. As Barassi and Treré (2012) remark, “the Web needs to be understood as an integrated socio-technical system, in which different Web applications and stages coexist” (p. 1273).

#### From digital information recipients to Indigenous digital negotiators and creators

The main feature of the Web 2.0 and 3.0 eras is moving beyond *Digital Information* to *Digital Negotiation* and *Digital Creation*. ILR initiatives have more widely implemented digital technologies with the view of enabling Indigenous language speakers and learners, in both remote and urban areas, to access informal, formal and self-directed language and cultural learning opportunities. These include using the Internet and web-based resources to share land-based planning activities, such as information about hunting, fishing and other traditional economic activities (Beaton & Carpenter, 2016). For example, SIKU (Sea Ice) is an Inuit Knowledge Wiki and Social Mapping Platform app which shares traditional knowledge information and satellite imagery to Inuit communities (Heath & Arragutainaq, 2019). And in Southeast Asia, the web-based eToro application, under the control of the Penan Indigenous community, stores traditional botanical knowledge in the Penan language and assists in passing traditional knowledge to the youth (Zaman et al., 2015). These small examples “connect youth and Elders to help promote intergenerational knowledge transmission . . . all the while encouraging language revitalization” (Winter & Boudreau, 2018, p. 45).

Digital and online spaces for learning and implementing Indigenous languages have also begun to move beyond viewing or clicking on materials to enabling more opportunities for more collaborative and multimodal interaction, negotiation and creation. For example, the use of keyboard, audio, video, screen and image. Incorporating the multimodality that advances in technology can afford also goes well with Indigenous ways of knowing and being. For example, the oral transmission of knowledge through storytelling and learning by doing (Battiste, 2002).

Websites and apps which are *facilitative*, such as dictionaries, verb conjugators, and spellcheckers; *collaborative*, such as games, forums, and simulations; and *instructional*, such as teaching materials and drills (Wagner, 2017), have been created. The Passamquoddy-Maliseet Language Portal (www.pmportal.org) is an example of a *facilitative* web collection of language documentation materials which contains short videos of conversations and interactions between fluent Passamaquoddy-Maliseet speakers (Wagner, 2017). KOBE Learn is an *instructional* app, developed by language teachers, Elders and community members in northern communities in Canada and designed to help young users learn common words and phrases in the Ojibwe, Cree and Oji-Cree languages (Hadley, 2019). Talk Sauk (www.talksauk.com) is a *collaborative* website, created by the Sauk language department in collaboration with Elders, which has an interactive dictionary, games, videos and more (Wagner, 2017). In addition, the latest innovation of FirstVoices, the BC language revitalization initiative highlighted in the Web 1.0 section, is a Keyboard App (www.firstvoices.com/content/apps) that enables users to type in over 100 Indigenous languages on any social media app or technological device. These initiatives are *low-tech* (one sensory mode), *mid-tech* (two sensory modes) and *high-tech* (multimodal interactive technology) (Galla, 2009), depending on community needs and the learning context.

#### Indigenous Internet creators decolonizing the digital landscape

In the Web 2.0 and Web 3.0 eras, Indigenous communities have gone beyond being recipients of information or collaborators to also being Internet “Produsers” (Kelly-Holmes, 2019) and creators. They have had more control and self-determination over the content produced and created (see Table 2). This self-determining creation step is necessary to decolonize the digital landscape and ensure that Indigenous voices and worldviews are also represented and privileged online in a culturally relevant way.

**Table 2.** Consolidation of Indigenous Internet creators decolonizing the digital landscape.

Technology use	Examples
Apps	SIKU KOBE Learn eToro
Websites	Passamquoddy-Maliseet Language Portal Talk Sauk FirstVoices
Movies, music and video games	Cellphilm (cellphone videos) SGaawaay K’uuna (*Edge of the Knife*) (movie) Never Alone; Honour Water (videogames) *Wolastoqiyik Lintuwakonawa* (music album)
Social media	Facebook Indigenous YouTube, Instagram, Twitter, TikTok videos
Coding	Pinnquaq Association Virtual Songlines Australia Coders North
Digital archives	C’ek’aedi Hwnax Alutiiq Museum Language Archive Plateau People’s Web Portal FirstVoices
VR, AR and AI	Biidaaban: First Light (VR) Buffalo Tongue (VR and AR) Ogoki Learning (VR app) Abtec (VR, AR and AI) Te Hiku Media (AI) Hua Kiʻi (AR and AI app)

AR: Augmented Reality; AI: Artificial Intelligence; C’ek’aedi Hwnax: Legacy House; eToro: Indigenous Penan Peoples’ Botanical Knowledge Management System; Hua Ki‘i : Hua ‘Ōlelo (word) and Ki‘i (image); SIKU: Sea Ice; Indigenous Knowledge Social Network; VR: Virtual Reality; Wolastoqiyik Lintuwakonawa: Our Maliseets Songs

Movie, video and song projects have been and are being developed by Indigenous Peoples with a focus on Indigenous languages and cultures. Cellphones and *cellphilm* (Schwab-Cartas, 2018) have been used to document and record embodied practices in an Indigenous language. For example, the filming of an Elder making traditional food in the Zapotec language in Mexico (Schwab-Cartas, 2018). Movies have been made in Indigenous languages which are critically endangered, such as the film SGaawaay K’uuna (*The Edge of the Knife*) released in 2018 and made entirely in the Haida language. And Jeremy Dutcher, the 2018 Polaris Music Prize winner, released the album *Wolastoqiyik Lintuwakonawa* (Our Maliseets Songs) in which Jeremy sings in the Wolastoqiyik language.

Video games are also providing a rich medium that reflects traditions of oral storytelling with different strategies for language and cultural preservation and revitalization (Lameman & Lewis, 2011). The Never Alone game was developed by the first Indigenous-owned gaming company, Upper One Games, in collaboration with the Iñupiat, an Alaska native people (Winter & Boudreau, 2018). Honour Water is a singing game and features Anishinaabe songs and teachings about the importance of protecting water (Hearne & LaPensée, 2017).

Indigenous social media use has become more influential and visible during the Web 2.0 and Web 3.0 eras. Although social media can have drawbacks and very real negatives such as cyber bullying and cyber racism, platforms such as Facebook, Twitter and YouTube have assisted ILR and Indigenous communities in sharing community and cultural knowledge, events, memes and snippets of language (Castleton, 2018; Rice et al., 2016). Sharing stories or videos online as part of Facebook groups, Instagram or Twitter posts, or on YouTube and TikTok enables Indigenous young people to be their Indigenous identities. Indigenous youth can connect, affirm and give a voice to their own particular cultural and linguistic identities which have not been constructed, imagined, or set by outsiders (Katsi’sorókwas Jacobs, 2019; Rice et al., 2016).

Indigenous coders and coding initiatives have become more prominent. The Pinnquaq Association piloted a coding workshop where Inuit children created their own sites and content (Toth et al., 2018). Virtual Songlines in Australia taught Indigenous youth to code their own content and be proud of their culture and heritage (Microsoft Asia News Center, 2018). Coders North in Northern Ontario also launched in 2019 to bring together Indigenous digital producers, teach coding and highlight opportunities for Indigenous youth to learn through digital technology (Engel, 2019).

More Indigenous-led and -guided digital archives and content management systems (CMS) are emerging as a response to colonizing effects of exclusion, discrimination and annihilation of Indigenous knowledges, Peoples and lifeways (O’Neal, 2014). C’ek’aedi Hwnax (Legacy House), established in 2009, “is the first OLAC [Open Language Archive Community] -compliant, Indigenously-administered digital language archive in North America” (Berez et al., 2012 p. 237). FirstVoices hosts Indigenous public and private community sites for language archiving where the Indigenous community members retain ownership of any content they create. Mukurtu (Dilly Bag) CMS is a “community driven software that addresses the ethical curation of, and access to, [Indigenous] cultural heritage” (Christen et al., 2017 para. 2). Mukurtu is used by various Indigenous communities and organizations globally for their digital language and culture archives, such as the Alutiiq Museum Language Archive in Alaska (www.languagearchive.alutiiqmuseum.org) and the Plateau People’s Web Portal (https://plateauportal.libraries.wsu.edu/). Although there is still much work to be done to safeguard Indigenous data sovereignty and ensure respectful, reciprocal and reconciliatory archival collections (O’Neal, 2014), these developments “exemplify how communities long regarded as objects of study have instead increasingly become leaders in the study and stewardship of their own languages” (Henke & Berez-Kroeker, 2016, p. 425).

Indigenous scholars, creators and visionaries are also making an impact in emerging AI, AR and VR technologies. Aboriginal Territories in Cyberspace (www.abtec.org) and the Initiative for Indigenous Futures (www.indigenousfutures.net) are Indigenous-determined research networks at Concordia University, Montréal, who are consolidating Indigenous presence in virtual worlds. Ogoki Learning develops immersive Indigenous language learning apps using VR (www.ogokilearning.com). Buffalo Tongue is an Indigenous-led non-profit which “create[s] virtual and augmented reality experiences to advocate for Native American voices, languages, and cultures” (Running Wolf in Lewis, 2020, p. 186). Bidaaban: First Light is a 2018 VR movie with narration in the Wendat, Mohawk and Ojibwe languages. Te Hiku Media, created from Indigenous language data and following cultural protocols, is able to deploy the “first speech-to-text algorithm in Te Reo Māori” (Moses in Lewis, 2020, p. 162). And Hua Kiʻi is an AR prototype of an “Indigenous language image recognition app with geolocation functionalities [which] allows the user to take a photo of an object and learn the word for that object” (C. Running Wolf et al., 2020, p. 110).

### Key takeaways and insights on the role of decolonizing technology from the present-day Web 2.0 and Web 3.0 eras

Digital and online technologies in the Web 2.0 and 3.0 eras are dramatically more inexpensive than in the Web 1.0 era and barriers to entry have been considerably reduced. The digital divide, despite still needing improvements in terms of physical and non-physical access and equitable representation, begins to narrow as most Indigenous Peoples, even in remote areas, now have access to a cellphone and use it to interact and communicate (Carpenter et al., 2017; Rice et al., 2016). Cellphones can record, film and connect to Internet. Indigenous Peoples no longer need to rely on governmental or external funding to start projects as many ILR initiatives can be started from the home community. For example, the AR prototype Hua Kiʻi “currently has a modest feature set but 10 years ago the technologies that enable it were unattainable beyond well-funded labs” (M. Running Wolf, 2020, p. 120).

The digital landscape continues to be decolonized. There are now more Indigenous technologies and learning environments that have been implemented and created by and for Indigenous Peoples. For example, Indigenous sites, software developers, coders, Indigenous AI, AR and VR. The Internet and digital technologies can foster a transnational space where colonial nation state binaries and linguistic boundaries are dissolved (Darwin & Norton, 2014). Indigenous Peoples can assert their “right to speak” (Darwin & Norton, 2014, p. 59) in their Indigenous languages and in a way that is respectful to their local communities.

Indigenous creators and technologists are counteracting negative and Western or colonial imposed stereotypes which view Indigenous Peoples as being confined to a very specific place, time and land (Winter and Boudreau, 2018). Stereotypes like this perpetuate a museological context (Castleton 2018); broadstroke Indigenous Peoples into false dichotomies, such as traditional as opposed to modern; and facilitate a colonial and imperialistic exploitation and conquest of the digital world. Kornai (2013), for example, in his case study *Digital Language Death*, claims that what is underway “is not just a massive die-off of the world’s languages, it is the final act of the Neolithic Revolution, with the urban agriculturalists moving on to a different, digital plane of existence, leaving the hunter-gatherers and nomad pastoralists behind” (p. 10). Not only is a racist remark of neo-Darwinist linguistic analysis and tradition (Macedo, 2019), his case study does not acknowledge that language *death* or *loss* is a byproduct of oppression and colonization (Bird, 2020). Kornai’s (2013) study also does not take into account endangered language cellphone use, apps or social media, such as Facebook or Instagram. These modes have been largely used, especially by Indigenous youth, in ILR initiatives during the Web 2.0 and 3.0 eras.

Indigenous Peoples decolonizing the digital landscape are breaking habits of algorithmic, linguistic and technological colonization (Bird, 2020). For example, Western linguists or scholars, such as Kornai (2013), can assume that some Indigenous languages are under-resourced due to a lack of textual speech data or standardized language forms. Bird (2020) remarks that this is a “colonising frame” which assumes “that major western languages are standard-bearers, and that Indigenous languages need the standard technologies” (pp. 3507–3508). For some Indigenous communities and ILR initiatives, the goal may not be fluency or to have standardized language forms, voice recognition, such as Siri and Alexa, spellcheckers, or autocorrect. As Leanne Hinton remarks, “language revitalisation in areas of language diversity and small populations is going to be very different than for languages like Hebrew, Hawaiian and Māori” (University of British Columbia, 2015). Bird underscores, “a dictionary app may represent an access point for one participant, and a marker of recognition in the digital realm to another. And this latter is no less valuable, given that prestige is a factor in language shift” (p. 3509).

More research needs to be carried out by or with Indigenous Peoples on how Indigenous Peoples view and use technology, what purpose this serves or has served, and whether or not this impacts on day-to-day language usage and promotion of cultural identity. Indigenous language learners need to find technology culturally engaging (Pitawanakwat, 2018) and integrated in an accessible, current and user-friendly way that enables language learning beyond isolated words or decontextualized phrases (Galla, 2009). The aim of many community ILR initiatives is to promote Indigenous language usage in domains such as the home or everyday social contexts (Hinton, 2003, 2013; Pitawanakwat, 2018). Technology-enabled and community-determined ILR (see Table 2) can assist in this process by moving beyond decontextualized Western learning objectives and embodying the “cultural, historical, ecological, and spiritual contexts that underlie the way a community defines its language” (Leonard, 2017, p. 18).

Many technology-enabled and self-determined ILR initiatives during the Web 2.0 and 3.0 eras have been centering community needs rather than externally defined or set goals, such as grammatical fluency (Leonard, 2017) or a digitally “thriving status” (Kornai, 2013, p. 1). Leonard (2017) calls this a framework of “language reclamation” which “centers community definitions of language at every stage, and thus prioritizes Indigenous needs and ways of knowing in the academic research, language pedagogies, and other work that underlie a given community’s language efforts” (p. 18). Technology use that is responsive to the local Indigenous community can foster more ethical relationships and “relational language technologies” (Taylor et al., 2019, p. 3511) going forward.

### Conclusion: Looking to the future of decolonizing technology in ILR

The role of technology in ILR has grown and evolved in a short space of time from being an extension of dominant Western hegemonies (Kelly-Holmes, 2019) to one in which Indigenous Peoples have an active and important voice in how technology is used, envisioned and created. Even if some underlying software is in English, Indigenous creators and developers are being “firm in applying Indigenous *thought* and *practice* into [the] . . . design and construction” (emphasis added, C. Running Wolf et al., 2020, p. 113).

Big questions regarding data sovereignty, for example, will always have to be asked before simply copy-pasting technology into ILR initiatives. Which system of knowledge is privileged? Where is the information or knowledge stored? Who has the power to access the knowledge and create streams of knowledge transmission? As illustrated in this non-exhaustive article, “Indigenous communities have long been engaged in the process of ensuring that technology platforms reflect and respond to their traditional ways, cultures and languages” (Carpenter et al., 2017, p. 10). This process continues to this day with Indigenous social media, websites, movies, music, apps and more. There are Indigenous Peoples working to ensure that the rapidly evolving future of AI has an ethical foundation rooted in and reflective of Indigenous worldviews and languages (Lewis, 2020).

The social use of technology in ILR is also not necessarily considered a substitute to real-life face-to-face interaction or as a panacea for ILR. Technology can be in relation to existing and future initiatives, a means to reclaim pride in Indigenous languages and cultures, and a way for existing and future speakers and learners to learn and interact. As with face-to-face interactions, the intent and relationship that one forms and builds with technology will decide what impact its present and future use will have. Warschauer (1998) remarked, “Can Indigenous peoples appropriate new network technologies for their own purposes, or in attempting to do so will they see their own cultures and languages swallowed up in a homogenous whole?” (pp. 139–140). More than 20 years and several stages of digital and online technologies later, some may ask the same question. The answer lies in no longer viewing culture or language as a static, decontextualized, monolithic entity; no longer measuring Indigenous Peoples or languages against colonial yardsticks; and Indigenous Peoples having complete self-determination over their use, negotiation, implementation, and creation of technology. This self-determination also means Indigenous communities can choose to engage “outside experts” in ILR projects based on their terms and needs (Bird, 2020, p. 3507).

This article demonstrates that there are very promising indicators of Indigenous socio-technological self-determination. Indigenous content creators, developers, and visionaries are becoming increasingly visible and influential in decolonizing the digital landscape to better serve Indigenous Peoples, their languages, and their communities.
